# Intracranial Hypotension with Multiple Complications: An Unusual Case Report

**DOI:** 10.1155/2013/913465

**Published:** 2013-11-13

**Authors:** Swetha Ade, Majaz Moonis

**Affiliations:** Department of Neurology, University of Massachusetts Medical Center, 55 Lake Avenue North, Worcester, MA 01655, USA

## Abstract

*Background*. Undiagnosed intracranial hypotension can result in several complications including subdural hematoma (SDH), subarachnoid hemorrhage (SAH), dural venous sinuses thrombosis (CVT), cranial nerve palsies, and stupor resulting from sagging of the brain. It is rare to see all the complications in one patient. Furthermore, imaging of the brain vasculature may reveal incidental asymptomatic small aneurysms. Given the combination of these imaging findings and a severe headache, the patients are often confused to have a primary subarachnoid hemorrhage. *Case Report*. We present a patient with spontaneous intracranial hypotension (SIH) who had an incidental ophthalmic artery aneurysm on MR imaging, and this presentation led to coiling of the aneurysm. The key aspect in the history “postural headaches” was missed, and this led to life threatening complications and unnecessary interventions. Revisiting the history and significant improvement in symptoms following an epidural blood patch resulted in the diagnosis of SIH. *Conclusion*. We strongly emphasize that appropriate history taking is the key in the diagnosis of SIH and providing timely treatment with an epidural blood patch could prevent potentially life threatening complications.

## 1. Introduction

Spontaneous intracranial hypotension (SIH) is often an underdiagnosed condition resulting from low cerebral spinal fluid (CSF) pressure. SIH usually presents with headaches following a dural sleeve tear resulting in CSF leak [1]. Headaches are typically orthostatic [2] but can also present as persistent daily headaches. Careful history taking is the key in making the diagnosis. Failure to diagnose may lead to life threatening complications including SAH, SDH, and CVT. We report a case of SIH, with all three complications described above. Our case is unique because, to the best of our knowledge, we have not come across any reported case of SIH with all the complications in one patient.

## 2. Case Report

**Figure 1:**
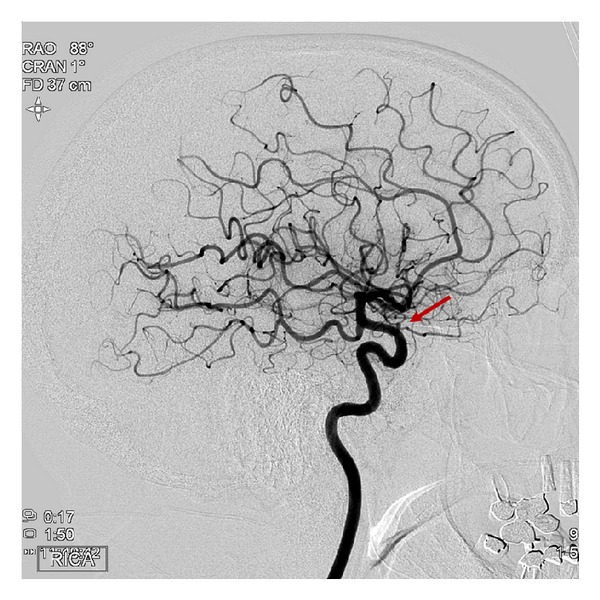
Cerebral angiogram showing 1.8 × 1.6 mm right ophthalmic artery aneurysm.

**Figure 2:**
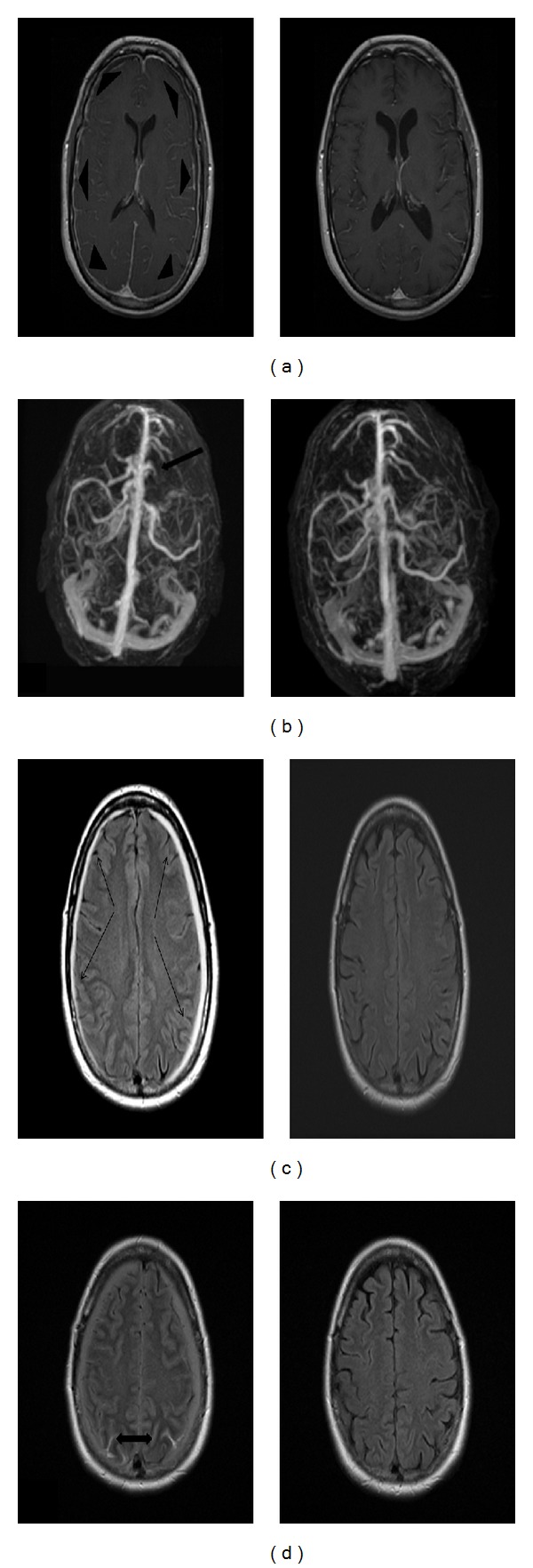
Right side (pretreatment) and left side (posttreatment). Axial T1 weighted magnetic resonance imaging (MRI) after intravenous administration of gadolinium. (a) Brain showing resolution of the pachymeningeal enhancement (arrow heads); (b) magnetic resonance venogram showing occlusion of the left cortical vein (arrow). Axial fluid attenuated inversion recovery MRI brain (c) showing resolution of subdural hematomas (arrows) on the bilateral convexities; subarachnoid hemorrhages (d) in the bilateral parietal lobes (bidirectional arrow).

A 54-year-old woman with history of migraines presented to ER with worst headaches of life for the past few days. Although she had a history of migraines, this headache differed from her usual migraines. In the ER subarachnoid hemorrhage (SAH) was suspected and a noncontrast head CT was done which was negative for any hemorrhage. As she continued to have severe headache, a lumbar puncture (LP) was done which was negative for xanthochromia, and thus SAH was ruled out. She was sent home with recommendation for over-the-counter analgesics. Following discharge patient continued to experience worsening of headaches associated with nausea, and vomiting. Three days later she presented to the emergency department (ED) with worsening headaches, nausea and hiccups preceding vomiting. Two days later MRI of the brain was done which revealed subdural fluid collections over the bilateral convexities and SAH in the parietal and occipital lobes. There were prominent cortical veins but no obvious CVT. At this point the etiology of the headache was unclear as the head CT during the initial ED visit was negative for any subdural or SAH. Because of venous engorgement, a cerebral venogram was done and was negative for venous sinus thrombosis. A four-vessel angiogram was performed to investigate the cause of SAH which revealed a 1.8 × 1.6 mm right ophthalmic artery aneurysm (Figure 1). Rupture of this ophthalmic artery aneurysm was presumed to be the cause of SAH and headaches. It was stented and coiled. However, the patient showed no improvement in symptoms over the next few days and a repeat magnetic resonance venogram (MRV) was done which revealed a cortical vein thrombosis on the left side with extension into the superior sagittal sinus. CVT was treated with anticoagulation therapy. The patient's headache, nausea, and vomiting continued. A repeat careful history revealed that she was comfortable lying down and headaches escalated upon standing. These headaches were different from usual migraines in that they were more positional, worse when sitting or standing. Reviewing the initial brain MRI, pachymeningeal enhancement and sagging of the pituitary fossa were noted, and hence SIH was suspected. Anticoagulation was reversed and a blood patch was performed which resulted in complete resolution of headaches, nausea, and vomiting. Follow-up MRI of the brain 3 months later showed complete resolution of the subdural collections, SAH, and CVT (Figure 2).

## 3. Discussion

Spontaneous intracranial hypotension is an underdiagnosed condition. It is extremely important that we always keep this condition in our differential when evaluating a case of headache. Our patient in this case had a history of migraines, but during her initial visit to the ED she clearly stated that her current headache does not resemble her usual migraines. At this point of time during the history taking, a key component—positional nature of the headaches—was missed and hence the diagnosis of spontaneous intracranial hypotension. As the initial head CT was negative for SAH, lumbar puncture was done to rule out xanthochromia and was negative. This lumbar puncture clearly worsened her positional headache causing her to return to ED. As the patient had headaches prior to the LP, the positional component was undermined and workup for other causes including CVT and aneurysms was considered. Pachymeningeal enhancement and downward displacement of the brain are the characteristic imaging findings in SIH [3]. Subdural fluid collections in SIH range from simple thin hygromas to massive subdural hematomas [4]. Not surprisingly, the MRI of the brain had revealed subdural hematomas on the convexities and SAH in the parietal and occipital lobes. Based on the MRI, it does not appear that the ophthalmic artery aneurysm was the cause of the SAH, as it was located distal to the site of SAH. It was an incidental [5] finding and the risk of rupture of small (less than 5 to 7) aneurysms is less than 1% per year [6]. Spontaneous intracranial hypotension was an established risk factor for CVT [7, 8] and was found in about 2.1% of population with SIH. As the patient continued to have headaches, a repeat careful history revealed the diagnosis further confirmed by MRI findings. Positional nature of the headaches was clearly missed on the initial ER visit and has led to a wrong suspicion for SAH, and hence the LP was done. LP in our patient has worsened the patient's preexisting positional component of the headache and has subsequently caused the complications including subdural hematomas, sagging of the brain, SAH, and incidental finding of the aneurysm which lead to coiling, CVT-putting the patient at risk of complications of anticoagulation. Patients with severe headaches often report to the ED where rapid automated algorithms exist and sometimes the obvious history that would clinch the diagnosis is not obtained. Hence, we strongly emphasize the importance of history taking as illustrated in our case.
